# Supplementary material for: Gene-flow from steppe individuals into Cucuteni-Trypillia associated populations indicates long-standing contacts and gradual admixture
Source: Sci Rep. 2020 Mar 6;10:4253. doi: 10.1038/s41598-020-61190-0 (PMC7060214; doi:10.1038/s41598-020-61190-0)
Supplement: Supplementary file 1 — Supplementary Figures and Information. [file 41598_2020_61190_MOESM1_ESM.pdf]

Gene-flow from steppe individuals into Cucuteni-Trypillia associated populations indicates long-standing contacts and gradual admixture

Alexander Immel, Stanislav Țerna, Angela Simalcsik, Julian Susat, Oleg Šarov, Ghenadie Sîrbu, Robert Hofmann, Johannes Müller, Almut Nebel and Ben Krause-Kyora

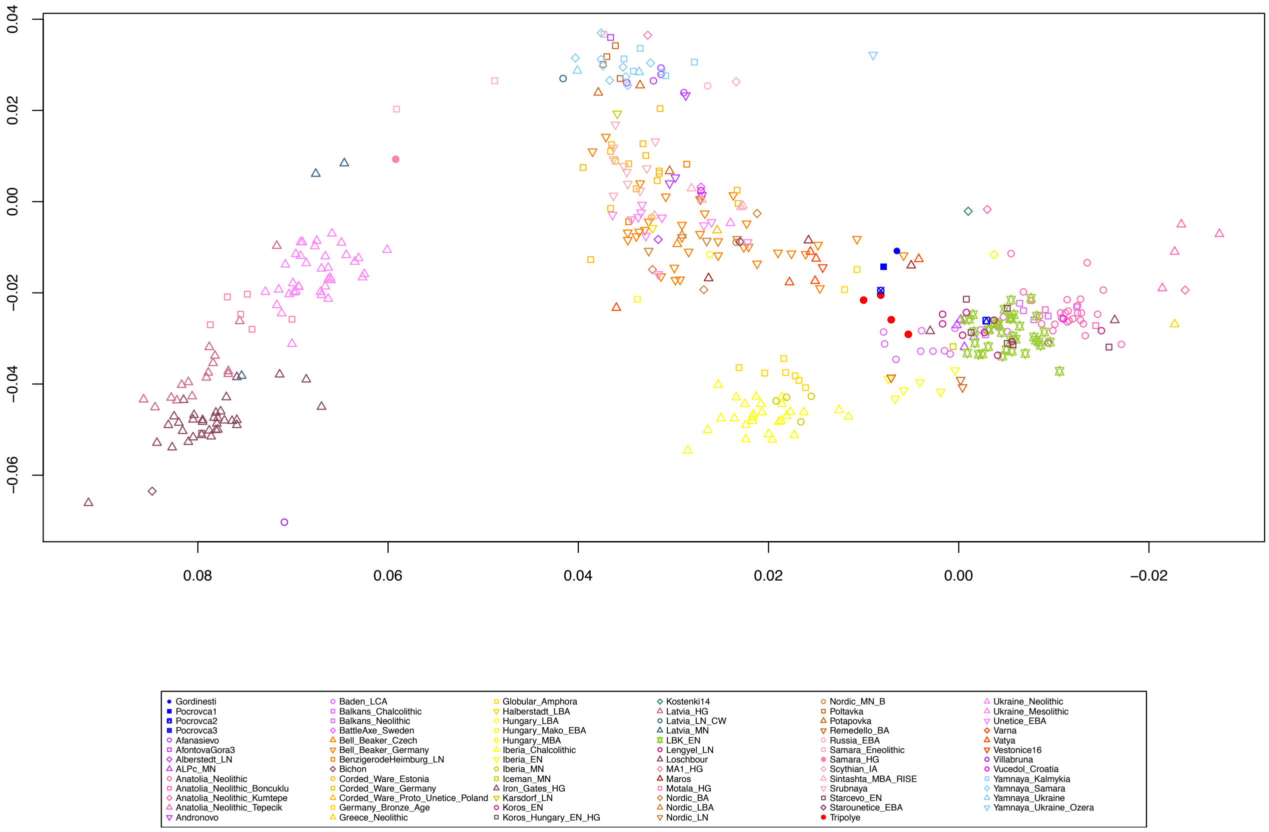

Supplementary Figure 1. Principal component analysis of the CTC individuals from Moldova (Gordinești, Pocrovca 1, Pocrovca 2, Pocrovca 3) in red and the CTC individuals from Verteba Cave (I1926, I2110, I2111, I3151) in blue together with 73 previously published ancient populations/individuals projected onto a basemap of 58 modern-day West Eurasian populations (not shown). EBA=Early Bronze Age, EN=Eneolithic, HG=hunter-gatherer, IA=Iron Age, LBA=Late Bronze Age, LBK=Linearbandkeramik, LN=Late Neolithic, M(L)BA=Middle (Late) Bronze Age, MN=Middle Neolithic, PU=Proto-Unetice, TRB=Trichterbecher (Funnel Beaker Culture, FBC). PC1 is shown on the x-axis and PC2 on the y-axis.

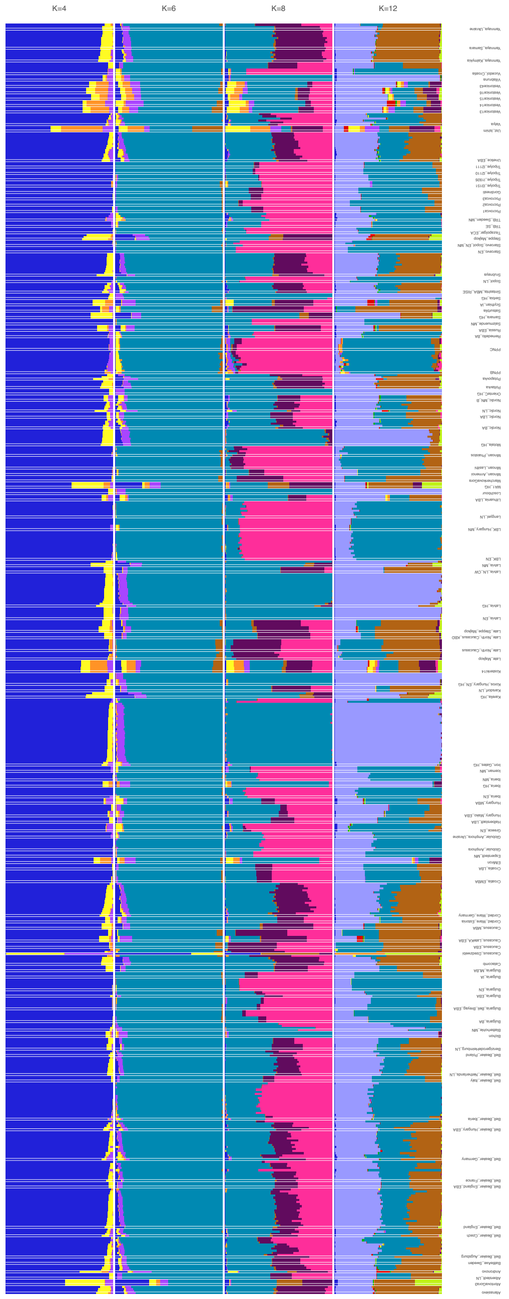

Supplementary Figure 2. Unsupervised admixture analysis for K=4, 6, 8 and 12 ancestral genetic components for 117 selected ancient populations/individuals. Modern-day populations were included in the analysis but are not shown. BA=Bronze Age, EBA=Early Bronze Age, EN=Eneolithic, HG=hunter-gatherer, IA=Iron Age, LBA=Late Bronze Age, LBK=Linearbandkeramik, LN=Late Neolithic, M(L)BA=Middle (Late) Bronze Age, MN=Middle Neolithic, PPNA=Pre-Pottery Neolithic A, PPNB=Pre-Pottery Neolithic B, PU=Proto-Unetice, TRB=Trichterbecher (Funnel Beaker Culture, FBC).

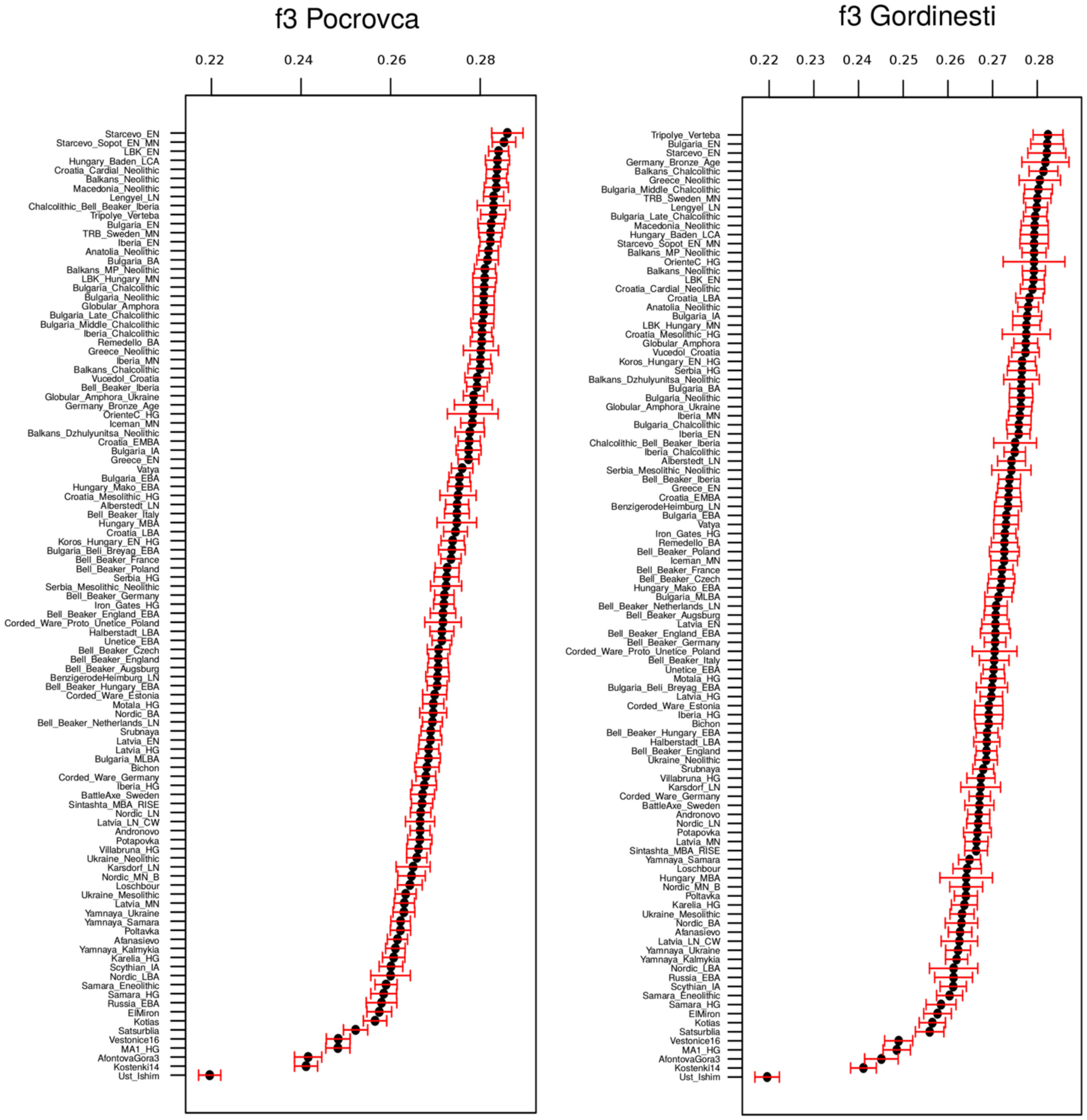

Supplementary Figure 3. f3-outgroup statistics *f3(Gordinești; Test, Mbuti)* and *f3(Pocrovca; Test, Mbuti)* showing the amount of shared genetic drift between our CTC individuals from Pocrovca and Gordinești, respectively, and previously published ancient populations/individuals. EBA=Early Bronze Age, EN=Eneolithic, HG=hunter-gatherer, IA=Iron Age, LBA=Late Bronze Age, LBK=Linearbandkeramik, LN=Late Neolithic, M(L)BA=Middle (Late) Bronze Age, MN=Middle Neolithic, PU=Proto-Unetice, TRB=Trichterbecher (Funnel Beaker Culture, FBC).

Skeletal Material

<u>KH160124: Pocrovka 5, pit 98 / 1998, skeleton 1/A, female, 60-65 years old (old adult)</u>

The preservation of the skeleton is medium to good. The skeleton is almost complete, found partially articulated.

Cranio-facial features: *calvaria*; very long, moderately wide and moderately high *neurocranium*; ovoid cranial vault shape (viewed from *norma verticalis*); dolichocrany; wide forehead; tall, moderately narrow and bulged occipital; moderately high facial skeleton.

Postcranial features: gracile bones; roundish humeri (euribrachy); hyperplatimeric femora (without pilastry); eurycnemic tibiae; average to tall skeletal stature (156-164 cm).

Anthropological type: Mediterranean and Dinaric features, in equal proportions.

Pathologies at the dental level: *antemortem* loss of 11 teeth from the lower arcade (partial mandibular edentation); advanced dental wear (attrition type).

Pathologies, non-metric traits and occupational stress markers at the cranial level: inactive porotic hyperostosis (cribrotic type *cribra cranii* and *cribra orbitalia*) on the occipital and parietals; small button osteoma on the frontal; complete supraorbital foramen (right side); frontal notch (left side); zygomatic multiple foramina (bilateral); postcoronal depression; very pronounced nuchal muscle attachments.

Pathologies, non-metric traits and occupational stress markers at the postcranial level: generalized degenerative osteoarthritis manifested through osteophytes, corrosion, eburnation, and deformed joint surfaces; spondylolysis of the fifth lumbar vertebra; squatting facets of the tibiae (more pronounced on the right side); additional femoral trochanter.

<u>KH160122: Pocrovka 5, pit 98 / 1998, skeleton 2/B, female, 20-25 years old (young adult)</u>

The preservation of the skeleton is medium to good. The skeleton is almost complete, found partially articulated.

Cranio-facial features: *calvaria*; very long, moderately wide and quite high *neurocranium*; ovoid cranial vault shape (viewed from *norma verticalis*); pronounced dolichocrany; moderately wide forehead; tall, moderately narrow and bulged occipital; moderately high facial skeleton; quite robust mandible.

Postcranial features: gracile bones; platybrachic humeri; platimeric femora (without pilastry); mesocnemic tibiae; above-average skeletal stature (156-160 cm).

Anthropological type: Mediterranean and Dinaric features.

Pathologies at the dental level: quite thin supragingival plaque on the upper and lower front teeth (on the vestibular and lingual sides); occupational / cultural dental wear (abrasion type) on the upper front teeth.

Pathologies and non-metric traits at the cranial level: inactive porotic hyperostosis (porotic type *cribra cranii*) on the occipital and parietals, more concentrated in the proximity of the sagittal and lambdoid cranial sutures; unilateral *foramen parietale* (left side); frontal notch (bilateral); double zygomatic foramina (left side).

Pathologies, non-metric traits and occupational stress markers at the postcranial level: hypertrophic periosteal reaction on the diaphyses of the tibiae and femora; *preauricular sulcus* (parturition scars); pronounced muscle attachments on the humeri and femora; additional femoral trochanter.

<u>KH160123: Pocrovka 5, pit 98 / 1998, skeleton 3/C, female, 35-40 years old (middle-aged adult)</u>

The preservation of the skeleton is medium to good. The skeleton is almost complete, found partially articulated.

Cranio-facial features: *calvaria*; long, wide and quite high *neurocranium*; ovoid-pentagonal cranial vault shape (viewed from *norma verticalis*); pronounced mesocrany; moderately wide forehead; tall, quite bulged and wide occipital; relative low facial skeleton; quite gracile mandible.

Postcranial features: gracile bones; platybrachic humeri; platymeric femora (with pronounced pilastry); platycnemic tibiae; average to above-average stature (154-158 cm).

Anthropological type: Mediterranean and Dinaric features.

Pathologies and non-metric traits at the dental level: supragingival plaque on the front teeth (vestibular side); occlusal and cemento-enamel junction (CEJ) cavities on the mandibular teeth; *antemortem* loss of two teeth not long before death; two apical abscesses; congenital absence of the third molar; moderate dental wear (attrition type).

Pathologies and non-metric traits at the cranial level: active porotic hyperostosis (cribrotic type *cribra cranii*) on the frontal, occipital and parietals; unilateral *foramen parietale* (right side); complete supraorbital foramen (left side); double zygomatic foramina (bilateral); intrasutural ossicles on the lambdoid cranial suture (left side).

Pathologies, non-metric traits and occupational stress markers at the postcranial level: hypertrophic periosteal reaction on diaphysis of humeri, radii, ulnae, femora, tibiae and fibulae; slight *preauricular sulcus* (scars of parturition); supratrochlear foramen of the left humerus; extremely pronounced and rugged *linea aspera*; femoral pilastry (bilateral); additional femoral trochanter; entheseal changes on the femoral muscle attachments.

<u>KH160121: Gordinești / 1981, grave 1, probably female, 9 years old ± 24 months (child or infans II)</u>

The preservation of the skeleton is medium to good. The skeleton is incomplete. The skull is represented through occipital, upper maxilla (partial), mandible (with deciduous and permanent teeth in the sockets and without symphysis), nine fragments from parietals, two from the temporals, one from the frontal (right side), one from the sphenoid and two isolated upper teeth (first and second right upper incisors). The postcranial skeleton includes the illia, a rib fragment and six cervical vertebrae (C1-C6).

The age at death was estimated by analysing the temporary/permanent dentition. Sex was determined by taking into consideration features of the entire skeleton, especially the illia and the skull fragments.

Pathologies at the cranial level: active porotic hyperostosis (cribrotic type *cribra crania* and *cribra orbitalia*) on the occipital, parietals and superior wall of the right orbit (left half of the frontal bone is missing).

<u>Taphonomic data</u>

The preservation of the remains is generally satisfactory. The skeletons of the adult females from Pocrovka V are almost complete, and the sub-adult skeleton from Gordineşti is partially represented (25-75%). The taphonomic analysis showed a very slight subaerial weathering on all remains (in form of thin calcareous depositions). No skeletal element presents traces of aerial weathering, such as cracking or peeling of the bone surface. All breakages and fissures that have led to the fragmentation of the skeletal elements occurred *postmortem*. No *perimortem* breaks were identified. All disarticulations were produced *postmortem*. There were no traces of slicing or scraping, chopping tool marks, impact / percussion marks, polishing or abrasion, or cut marks from defleshing activities. It is important to mention that there are no elements that indicate decapitation. Carnivore-ravaged marks or rodent tooth scratches, gnawing, and tooth punctures are missing. Root-etching on the

bone surfaces is not very evident and is present only on some skeletal elements, forming a discrete dendritic network. Burning traces are missing. The body parts were decomposed *in situ*.

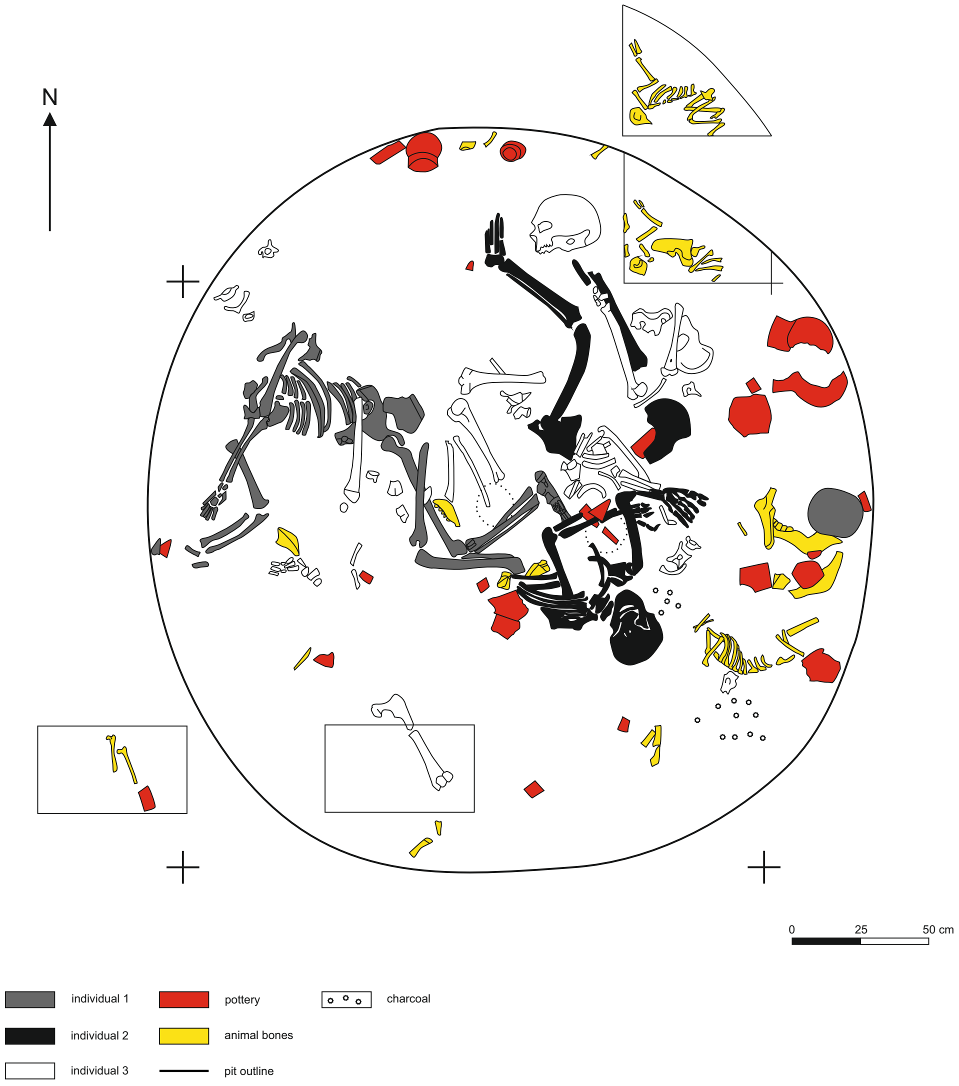

Plan of the bottom of pit 98 from the Pocrovca V settlement with the three human skeletons which provided genome-wide data for the study (drawing O. Šarov; digitalization S. Țerna).

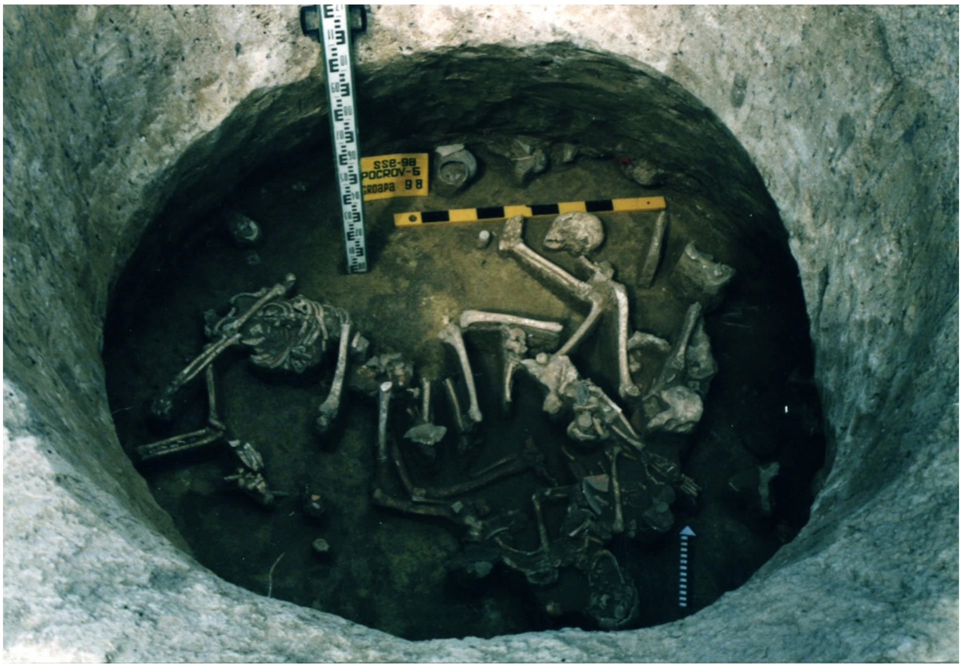

Photograph of the bottom of pit 98 from the Pocrovca V settlement with the three human skeletons which provided genome-wide data for the study (photo O. Šarov).
